# Graphene growth on Ge(100)/Si(100) substrates by CVD method

**DOI:** 10.1038/srep21773

**Published:** 2016-02-22

**Authors:** Iwona Pasternak, Marek Wesolowski, Iwona Jozwik, Mindaugas Lukosius, Grzegorz Lupina, Pawel Dabrowski, Jacek M. Baranowski, Wlodek Strupinski

**Affiliations:** 1 Institute of Electronic Materials Technology, Wolczynska 133, 01-919 Warsaw, Poland; 2 IHP, Im Technologiepark 25, 15236 Frankfurt (Oder), Germany; 3 Department of Solid States Physics, University of Lodz, Pomorska 149/153, Lodz, 90-236, Poland

## Abstract

The successful integration of graphene into microelectronic devices is strongly dependent on the availability of direct deposition processes, which can provide uniform, large area and high quality graphene on nonmetallic substrates. As of today the dominant technology is based on Si and obtaining graphene with Si is treated as the most advantageous solution. However, the formation of carbide during the growth process makes manufacturing graphene on Si wafers extremely challenging. To overcome these difficulties and reach the set goals, we proposed growth of high quality graphene layers by the CVD method on Ge(100)/Si(100) wafers. In addition, a stochastic model was applied in order to describe the graphene growth process on the Ge(100)/Si(100) substrate and to determine the direction of further processes. As a result, high quality graphene was grown, which was proved by Raman spectroscopy results, showing uniform monolayer films with FWHM of the 2D band of 32 cm^−1^.

It is an irrefutable fact that the best quality graphene in terms of structural integrity and electrical properties is obtained by mechanical cleavage of highly oriented pyrolytic graphite^1^. Although pristine graphene has a very low concentration of structural defects, the flake thickness and location are largely accidental. Moreover, the size is limited by the single crystal grains in the starting graphite, thus being of the order of micrometers. Several strategies are presently being pursued to achieve reproducible and scalable graphene on various substrates. One example is graphene’s manufacturing technique based on conversion of SiC (0001) to graphene via sublimation of silicon atoms from SiC surface at high temperatures^2^,^3^. SiC crystals are also used as substrates to perform Chemical Vapor Deposition (CVD) growth of epitaxial graphene^4^. Graphene on SiC is beneficial since SiC is a well-established substrate for high frequency electronics, light emitting devices, and radiation hard devices. However, the size of the initial SiC wafer (usually not larger than 4”) is relatively small for applications based on large format graphene. Additionally, a very high temperature of the graphene growth process on SiC substrates (around 1600 °C) requires specialized equipment.

Recently, single crystal hexagonal boron nitride (h-BN) has been proposed as an ideal substrate for epitaxial growth of graphene^5^. Furthermore, it has been demonstrated that h-BN is an excellent substrate for electrical transport devices fabricated from exfoliated graphene flakes^6^. Nevertheless, treating it as a perfectly matched substrate leaves much to be desired when it comes to its size and controllable distribution on the applied substrates.

Lately, among many interesting methods developed worldwide^7^,^8^,^9^, CVD has been treated as one of the most auspicious, relatively cheap and readily available approaches to deposition of reasonably high quality graphene on non-carbide forming transition metal substrates such as Ni^10^,^11^,^12^, Pd^13^, Ru^14^,^15^, Ir^16^, Pt^17^,^18^,^19^, Cu^20^, Co^17^. Among various substrates on which graphene can be synthesized a copper substrate seems to be the most versatile and suitable one. The first report on growing graphene on copper foil was prepared by Ruoff^21^. The Authors have achieved high quality graphene films on copper foil, and finally have fabricated a dual-gate FET device.

Since graphene and its extraordinary properties were described and analyzed in depth, it has been considered as a material to be implemented in various fields. One of the leading ideas which appears to be a progressive concept is to apply graphene to complementary metal oxide semiconductor technology (CMOS). In order to widen the range of possible graphene applications, for example in high frequency electronics, it is desirable to grow graphene films directly on arbitrary insulators or semiconductor surfaces instead of on a most commonly used copper substrate. Ideally such insulating or semiconducting layers are deposited on Si wafers used commonly in integrated circuit (IC) manufacturing. Growing graphene directly on Si wafer surface is extremely challenging due to the tendency to form carbides. This forces researchers into looking for alternatives, for instance offered by germanium substrates^22^. In their case carbide formation does not appear and in consequence, growth as such is feasible. What is more, germanium technology is compatible with CMOS technology, and therefore there is no need for introducing any changes to the already existing technology. Additionally, in order to create a device structure one can perform selective growth of germanium layers and thereby grow graphene on a predefined device area. These advantages were exploited to grow high quality graphene on Ge(110)/Si(110) wafers^23^. Nevertheless, CMOS technology based on Si(100) wafers and this particular crystallographic orientation belong to the mainstream of Si IC fabrication.

In this paper, we intend to present, for the first time, CVD growth of graphene on epitaxial Ge(100) layers deposited on Si(100) wafers, which is the preferred wafer orientation in CMOS technology. The cost advantage offered by this approach is linked with the bulk buying of wafers and what is even more important manufacturing compatibility of Si(100). Additionally, when compared with techniques described in other reports^23^ our method does not require any special pretreatment of Ge(100)/Si(100) wafers such as *ex-situ* removal of native oxides or preceding graphene growth with the deposition of fresh Ge layers, which, without a doubt adds value to the fabrication process. As an alternative to direct growth, graphene transferring from a copper substrate is considered. Ruoff *et al.*^21^ showed that data obtained from a dual-gate FET device suggest that graphene films grown on Cu foil are of a reasonable quality, which is sufficient to encourage scientific society to continue improving the growth process to achieve a material quality equivalent to that of natural exfoliated graphite. However, one should be aware of contamination of copper associated with graphene transfer from Cu and present in the transferred layers^24^. Since CMOS technology requires as high material purity and stringent contamination control as possible, we aimed at a high quality of graphene transferred from Ge(100)/Si(100) structure avoiding the metal contamination problems. Obtaining high quality graphene grown on Ge(100)/Si(100) wafers which can be subsequently either transferred using the wafer bonding approach or used directly in the fabrication of e.g. graphene THz devices is essential^25^, thus opening the way for this new material to get integrated with Si microelectronics. In this paper we present how to obtain large area graphene in a reproducible way directly on Ge(100)/Si(100) substrates. In our experiment we attempted to grow graphene on solid Ge films instead of melted substrates^22^, which guarantees full control over the growth process and brings additional benefits such as feasibility of reusing substrates, easiest characterization, smoother surface as well as, possibility of transferring graphene^26^.

Additionally, in the literature there are examples of graphene growth modeling; however, the vast majority of these papers are related to graphene synthesis on substrates other than Ge(100)/Si(100), including mostly copper foils^27^,^28^,^29^. Nevertheless, there is also a need to model the graphene growth process on Ge(100)/Si(100) substrates because this can provide useful information on the control and optimization of the graphene growth process, which can be especially vital for the industrial-scale production. Therefore, the stochastic model is applied in order to describe the graphene growth process.

## Result and Discussion

In order to determine and understand the kinetics of the graphene growth process on the Ge(100)/Si(100) substrate, the nucleation stage was investigated as a function of methane flow. During the processes different methane flow were introduced into the growth chamber. The processes were performed in such a way that the results of the initial steps of graphene growth were observed. In other words, the processes were stopped before graphene formed continuous films and, hence, graphene flakes rather than continuous films were visible on the surface of Ge(100)/Si(100). Next, after having obtained a set of samples partially covered by graphene, a round of longer processes was completed with the aim of observing the formation of a continuous graphene film and the accompanying side effects like discontinuities, grain boundaries or secondary (2ML, 3ML or higher) graphene ad-layers.

**Figure 1:**
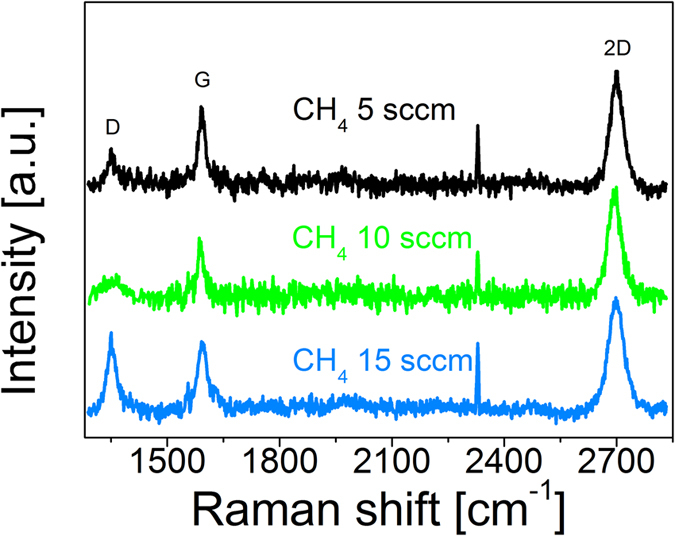
Raman spectra for 3 different methane flow settings.

**Figure 2:**
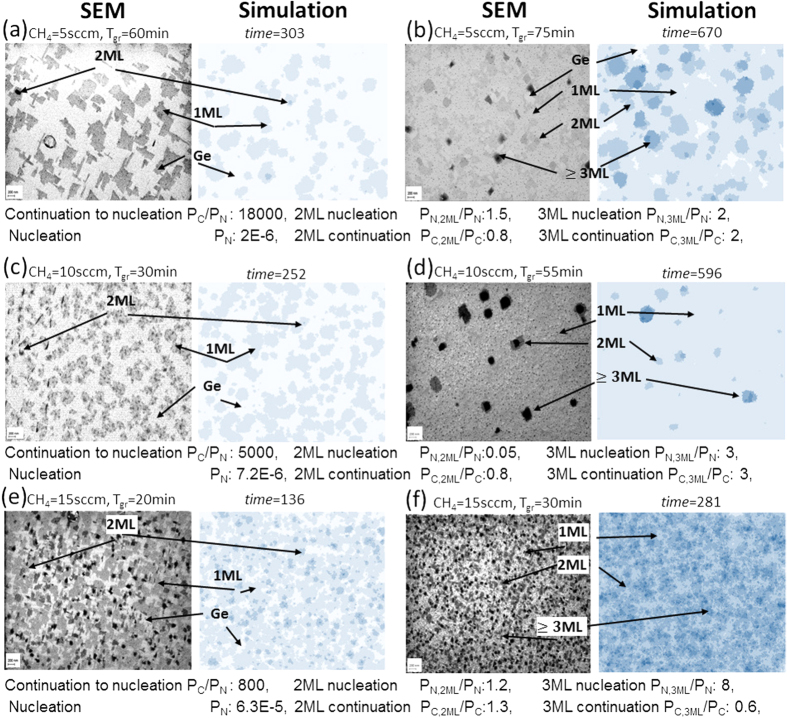
SEM images and simulation images for samples grown on Ge(100)/Si(100) with CH_4_ flow of (**a**) 5 sccm graphene flakes, (**b**) 5 sccm continuous film, (**c**) 10 sccm graphene flakes, (**d**) 10 sccm continuous film, (**e**) 15 sccm graphene flakes (**f**) 15 sccm continuous film. Scale bar is 200 nm. The simulation parameters are indicated below for each pair of growths. The simulations were conducted on regular lattice frame of 500 × 500 unit cells.

At the first attempt the Raman quality was investigated as a function of methane partial pressure. As for the preset growth parameters, CH_4_ flow varied from 5, through 10, up to 15 sccm (standard cubic centimeters per minute). The collected Raman spectroscopy results confirm the presence of graphene layers in the case each of the examined samples (Fig. 1). Widened 2D bands FWHM (Full Width of Half Maximum) of 2D bands is 50 cm^−1^, 42 cm^−1^ and 42 cm^−1^ for 15, 10 and 5 sccm, respectively) relative to free-standing graphene indicate that on the surfaces of the samples more than one layer of graphene was obtained^30^. However, the 2D/G intensity ratios (2.4, 2.1 and 1.8 for 5, 10 and 15 sccm, respectively) suggest that the monolayer of graphene is present on the surface as well^31^,^32^. This observation is easier to understand when one takes into account that the Raman spectrometer laser spot diameter on the samples surface was approximately 0.3 μm so during the measurement the laser spot enabled detection of graphene from areas containing one and a few layers. This co-existence of mono and multi-layers was also clearly seen in SEM images (Fig. 2b,d,f). Dark features identified as nuclei of multilayers of graphene were observed on a monolayer of graphene. The relatively high I_D_/I_G_ ratio equal to 0.4, 0.57 and 0.76 for 5, 10 and 15 sccm, respectively can be accounted for by two factors, namely the distance between isolated defects or the existence of very small graphene monocrystalline domains of the order of 40 nm^33^,^34^.

There is a direct correlation between carbon transport (as methane) and the growth rate of graphene flakes. The methane flow can also imply the nucleation rate (i.e. the number of new flake nucleations per time unit). In the simplest view, if growth is mass transport-limited, both values (growth rate and nucleation rate) should be close to proportional to the precursor partial pressure. In such a case, the precursor flow value does not affect the overall growth pattern of flakes, except the timescale. Several factors can disrupt this image. Nucleation can be quickly saturated by a finite number of nucleation defects on the substrate surface, as a result of which the growth rate increases faster than the nucleation rate. On the other hand, nucleation can get more efficient when the precursor flow is increased because of the limited migration of surface ad-atoms. The higher the precursor partial pressure, the shorter the time for single ad-atom to migrate and attach to a flake edge before getting companions and nucleating a new flake (even without any nucleation defects).

SEM images (Fig. 2a,c,e) show that the patterns of graphene flakes grown on Ge(100)/Si(100) substrates with the CH_4_ flow equal to 5, 10 and 15 sccm are different and the relationship between the growth rate and the nucleation rate was not the same for various CH_4_ flows. In each pair of images (SEM and simulation) different areas are marked with arrows. The varied areas in bluescale in simulation pictures correspond to the number of graphene layers (1ML, 2ML, 3ML, 4ML) seen in SEM images and the lighter blue areas in simulation images represent uncovered Ge surface, without graphene on top. Pictures suggest that the nucleation rate increases faster than the growth rate with raising the CH_4_ flow. Besides, the images show some secondary (2ML) or even 3ML or 4ML nucleations overgrowing the first layer (1ML). These ad-layers seem to nucleate and to increase their size with a highly varying rates, when comparing all three pictures.

In order to provide a more precise quantitative interpretation of both the growth statistics and the observed growth patterns of graphene on Ge(100)/Si(100) substrates we have developed a Monte-Carlo type model of the layered growth. The model employs two primary parameters, *P*_*N*_ and *P*_*C*_, as follows:

1. *P*_*N*_ is the probability of growing the graphene unit cell in coordinates of a single unit cell (on the material below) per time unit, if it does not have any already grown closest neighbours – the “nucleation” case.
2. *P*_*C*_ is the probability of growing the graphene unit cell in coordinates of a single unit cell per unit time, if its closest neighbour has been already grown – the “continuation” case.

The later used convenient parameter is a ratio of these two probabilities (*P*_*C*_* /P*_*N*_). The employed term “unit cell” assigns an arbitrary unit lattice area, which in the limiting case can be the crystal lattice unit cell. When more than one nearest neighbour is already grown, the growth probability can be simply set as the additive function of these neighbours.

The model applies a pair of secondary parameters: 1) the probability ratio of secondary nucleation on the already grown graphene layer to nucleation on the substrate - (*P*_*N,2ML*_*/P*_*N*_), 2) the probability ratio of continuation on the graphene layer to continuation on the substrate - (*P*_*C,2ML*_*/P*_*C*_). These factors describe the formation of the ad-layers. If analogous parameters were required for the description of higher order (ad-) ad-layers, they would be denoted as (*P*_*N,3ML*_*/P*_*N*_), (*P*_*C,3ML*_*/P*_*C*_), (*P*_*N,4ML*_*/P*_*N*_) or similar.

The simulation process consisted of repeating the following sequence: 1) Randomly selecting coordinates of a unit cell, 2) Checking the growth state of the nearest neighbour unit cells and assigning the growth probability value, 3) Drawing the occurrence of a given growth step event. The calculations were usually completed for the square lattice frame of 1000 × 1000 or 500 × 500 unit cells. The computed image of the primary (1ML) pattern (the covered area share and total number of nucleations per frame) does not depend on the calculation frame dimension, when scaling the parameters as below.


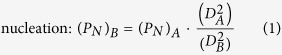



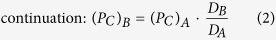


where *D* is the calculation image frame dimension (in unit cells), whereas *A* and *B* assign two calculation setups. If the *P*_*C*_* /P*_*N*_ ratio and image dimension are fixed, the image primary pattern does not depend on the *P*_*N*_ absolute value, when scaling the time as below:


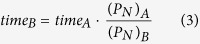


In conclusion, the primary (1ML) image is defined by only two values. The first is the function of time describing progress in the growth process: 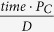. The second is the function of the *P*_*C*_* /P*_*N*_ ratio and the image dimension *D* describing growth. It is easy to show that this function is *a* quotient: 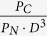. However, this low number of degrees of freedom can be seen as an introductory configuration of the considered model.

Several attempts to fit the microscope images of graphene on Ge(100)/Si(100) substrates with simulation have quickly given quite satisfactory patterns. Below (Fig. 2) there are results for three analysed pairs of growths (the only difference within a pair is the process time). These simulations were completed on a regular lattice with a calculation frame of 500 × 500 unit cells. Below each pair of images, relevant simulation parameters are indicated. The specified *P*_*N,..*_ and *P*_*C,..*_ values correspond to the probabilities of the occurrence of growth of a graphene unit cell after single drawing of the coordinates of this unit cell (in a single sampling of this cell). The “*time*” value corresponds to the average number of samplings of a single unit cell (the total number of samplings divided by the total number of unit cells).

The fitting results show that the (*P*_*C*_* /P*_*N*_) ratio decreases significantly with increasing the CH_4_ partial pressure – the continuation of the existing flakes becomes less preferred to nucleation of the new ones. Faster raising of the nucleation rate than the lateral growth rate with CH_4_ flow suggests that the nucleation is suppressed by some process, especially at a low precursor partial pressure. The most obvious explanation is as has been mentioned earlier: nucleation can be prevented by the migration of ad-atoms to the nearby flakes; when increasing the precursor flow, the time, in which ad-atom is lone, shortens. Before reaching a flake, the ad-atom transits into the ad-atom cluster. The clustering slows or stops migration and results in the nucleation.

The next parameter (*P*_*N,2ML*_*/P*_*N*_), which is related to the secondary (2ML) layers, does not exhibit a monotonic behaviour. The 2ML nucleation ratio (*P*_*N,2ML*_*/P*_*N*_) and even directly the nucleation rate alone (*P*_*N,2ML*_) shows a strong minimum around the medium CH_4_ flow, while outside this minimum they depend on the CH_4_ flow in the same manner as in the case of the primary nucleation – the *P*_*N,2ML*_*/P*_*N*_ ratio is almost constant there. We cannot offer a clear explanation for this effect yet. Such explanation would have to distinguish between the Ge(100)/Si(100) surface area and the 1ML graphene layer area as a substrate. It can, however, be supposed that the clustering process of ad-atoms includes an intermediate step during which they become exceptionally mobile on the graphene 1ML surface (while this does not take place on other surfaces) and this intermediate step is preferred by the medium precursor flow through a surface or by a gas phase reaction. Despite these differences in nucleation, the growth continuation parameter *P*_*C,2ML*_*/P*_*C*_ regarding the secondary (2ML) graphene layer does not significantly depend on the CH_4_ partial pressure and is close to *P*_*C,2ML*_*/P*_*C*_ = 1. It means that the continuation of flakes has a similar growth rate for both 1ML and 2ML flakes.

The nucleation rate of the higher secondary layers (3ML, etc.) seems to grow monotonically with the CH_4_ flow and to grow faster than the 1ML nucleation rate, the *P*_*N,3ML*_*/P*_*N*_ ratio reveals a significant increase. This can be seen as the secondary (2ML) flakes are very often overgrown by next-order layers, more often if the CH_4_ flow is higher. While the nucleation rate of higher-order (>2ML) ad-layers grow faster than the 1ML nucleation rate, the continuation rate seems to be more similar to the 1ML continuation rate.

The relation between the experimental growth time (T_gr_) and simulation *time* parameter within growth pairs is not directly proportional. This affected correlation and confirms the existence of a time offset before the nucleation becomes visible. The delay was clearly observed in other growth experiments, not shown in this paper. This offset partially results from the specific operation of the hardware; however, in its larger part it has to be induced by another factor. Most probably, it is related to the nucleation stage of 1ML graphene on Ge(100)/Si(100). The nucleation cluster with just a few atoms collects subsequent atoms much slower than a flake, until getting a regular lattice and becoming visible in SEM images.

To conclude, the growth simulation analysis leads to three distinct conclusions. Firstly, the nucleation rate of graphene on Ge(100)/Si(100) is affected by the migration of ad-atoms on the surface and this nucleation can be increased by accelerated clustering of ad-atoms in a high precursor partial pressure. Secondly, there is a striking difference between the first ad-layer nucleation on graphene (2ML) and primary nucleation on germanium since the ad-layer (2ML) nucleation rate has a strong minimum for a particular precursor flow. Thirdly, the nucleation, as such is a relatively slowly progressing step, introducing a time delay before substantial covering by graphene begins.

**Figure 3:**
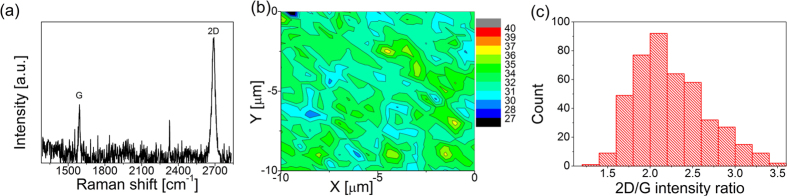
(**a**) Raman spectrum, (**b**) micro-Raman map of FWHM of 2D band and (**c**) histogram of 2D/G intensity ratio of optimized graphene film on Ge(100)/Si(100) substrate.

On the basis of the above mentioned results further optimization steps in the growth process of graphene on Ge(100)/Si(100) substrates were made. The methane flow was reduced and the time of growth was extended. As a result, a uniform graphene film was achieved. Figure 3 shows a Raman spectrum, micro-Raman map of FWHM of the 2D band and histogram of 2D/G intensity ratio for the obtained graphene layer. One can notice that there is no D peak in the spectrum. In addition, highly uniform, narrow 2D bands (32 cm^−1^) and the 2D/G intensity ratio higher than 2 are observable, which confirms the presence of a high quality and homogeneous monolayer graphene film.

**Figure 4:**
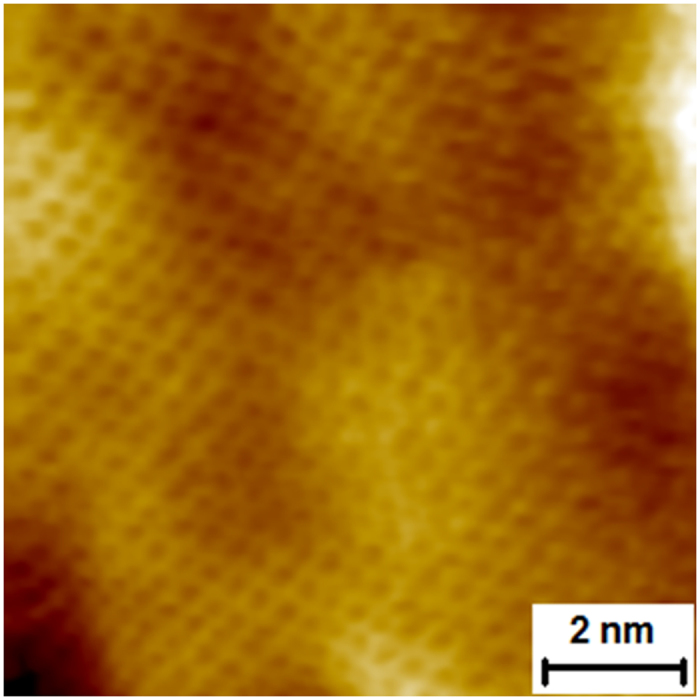
Atomic resolution STM of graphene on a Ge(100)/Si(100) substrate.

Additionally, in order to confirm the presence of the high quality monolayer of graphene on Ge(100)/Si(100) substrates STM measurements were performed. Figure 4 shows the atomic-resolution STM image of graphene on the said substrate. The characteristic honeycomb structure of graphene can be observed and single carbon atoms can be distinguished.

## Conclusions

In summary, the influence of the methane flow on the graphene nucleation mechanism, growth rate, uniformity and structural quality has been investigated. High quality graphene has been grown on Ge(100)/Si(100) substrates, which has been mainly confirmed by the results of Raman spectroscopy measurements. Also, the presented simulation and quantity analysis show that graphene growth on Ge(100)/Si(100) substrate can be described by a simple stochastic model with a very low number of parameters. The influence of the substrate lattice structure, extended defects and growth anisotropy are restrained enough to make such a description possible. The excellent alignment between the experiment and the simulation means that in the course of further investigation a convincing physical model can be proposed to describe the whole process of graphene growth. The presented attempt, including particularly direct growth on the Ge(100)/Si(100) substrate combined with modelling of the growth process, was shown here as a viable alternative enabling the introduction of graphene with its unique features to CMOS technology.

## Methods

### Sample preparation

In the present work graphene films were synthesized in a 6-inch Aixtron Black Magic system by the CVD method. As a substrate we used (100)-oriented Ge layers deposited by the CVD method on Si (100) wafers. To ensure optimal temperature conditions, thus preventing Si diffusion through a Ge layer and Ge melting, the temperature was monitored and set separately and simultaneously in bottom and top heaters. The temperature was chosen in the range between 900 °C and 930 °C with a temperature ramp up rate of 20 °C/min. During the process of graphene deposition the pressure range of 700 to 780 mbar was sustained. Methane gas was used as a carbon precursor in the mixture of Ar and H_2_ in the ratio of 20:1. It was found that the optimal roughness of the substrate is achieved when the step growth is preceded by the substrate’s annealing at pure hydrogen atmosphere in order to *in-situ* reduce native oxides^35^.

### Characterizations

The characterization of the properties of graphene grown directly on Ge(100)/Si(100) substrates was performed by Raman spectroscopy using a Renishaw system with a 532 nm Nd:YAG laser as an excitation source with the laser spot diameter on the sample surface of approximately 0.3 μm. The surface of graphene was investigated using the In-Lens secondary electrons detector (true SE1) and the Energy selective Backscattered electron (EsB, low-loss BSE) detector, both positioned on the optical axis of the Gemini™ column of the Auriga CrossBeam Workstation (Carl Zeiss). The energy of primary electrons in the scanning beam was set at 500 eV so as to reveal the morphology of the ultra-thin layer of graphene (single layer) and simultaneously distinguish different phases present on the substrate based on the compositional contrast (low-loss BSE). STM measurements were conducted under base pressure of 2 × 10^−10^ mbar with a Multiprobe P system made by Omicron GmbH.

## Additional Information

**How to cite this article**: Pasternak, I. *et al.* Graphene growth on Ge(100)/Si(100) substrates by CVD method. *Sci. Rep.* **6**, 21773; doi: 10.1038/srep21773 (2016).
